# Stereotactic posterior midline approach under direct microscopic view for biopsy of medulla oblongata tumors: technical considerations

**DOI:** 10.1007/s00701-020-04600-6

**Published:** 2020-10-13

**Authors:** Janine-Ai Schlaeppi, Lukas Andereggen, Andreas Nowacki, Claudio Pollo

**Affiliations:** 1 Department of Neurosurgery, Bern University Hospital, Inselgruppe AG, Freiburgstrasse 8, 3010 Bern, Switzerland; 2 Department of Neurosurgery, Kantonsspital Aarau, Tellstrasse 8, 5001 Aarau, Switzerland

**Keywords:** Stereotactic biopsy, Medulla oblongata, Brain stem, Glioma

## Abstract

**Background:**

Open and stereotactic transfrontal or transcerebellar approaches have been used to biopsy brainstem lesions.

**Method:**

In this report, a stereotactic posterior and midline approach to the distal medulla oblongata under microscopic view is described. The potential advantages and limitations are discussed, especially bilateral damage of the X nerve nuclei.

**Conclusion:**

This approach should be considered for biopsy of distal and posterior lesions. We strongly recommend the use of direct microscopic view to identify the medullary vessels, confirm the midline entry point, and avoid potential shift of the medulla. Further experience is needed to confirm safety and success rate of this approach.

**Electronic supplementary material:**

The online version of this article (10.1007/s00701-020-04600-6) contains supplementary material, which is available to authorized users.

## Relevant surgical anatomy

**Fig. 1:**
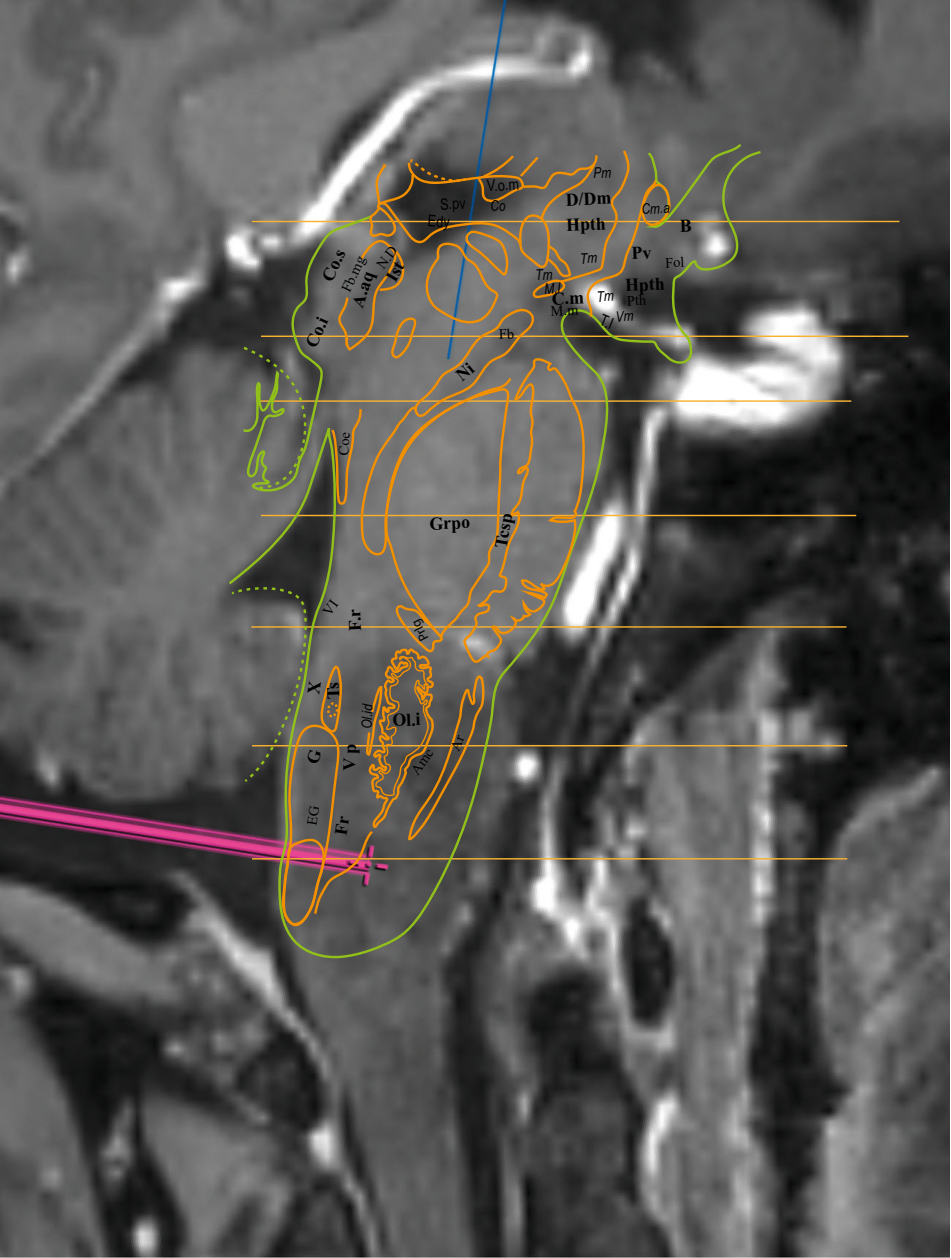
The Schaltenbrand and Wahren stereotactic atlas was superimposed and fused with the preoperative stereotactic trajectory planning, emphasizing the relation between the target being 43 mm below the X nerve nuclei

Stereotactic biopsy of the medulla oblongata is a rare procedure and remains challenging. The lower the lesion is located in the brainstem, the greater is the risk and severity of associated complications [1]. Because of the distal and posterior location of the lesion, a transcerebellar approach would not have been possible. A stereotactic posterior approach through the midline allows for the shortest trajectory through the brainstem. The stereotactic conditions provide the highest precision in reaching the target point through a precisely defined trajectory. The craniocaudal level of the entry point in the brainstem is crucial as the position of the X cranial nerve nuclei is located immediately parasagittal in the upper part of the medulla oblongata. According to the Schaltenbrand and Wahren stereotactic atlas [3], the caudal extension of the X nuclei is 33 mm below the zero point, set at the pontomesencephalic junction. Since the lesion was located in the lower part of the medulla oblongata in this case, our entry point was located 43 mm under the referential (Fig. 1) and was considered safe with this respect. The other relevant anatomical challenge is to ensure that the entry point is accurately located at the midline to avoid injury of posterior fiber tracts and to avoid injury of the brainstem superficial vessels.

## Description of the technique

**Fig. 2:**
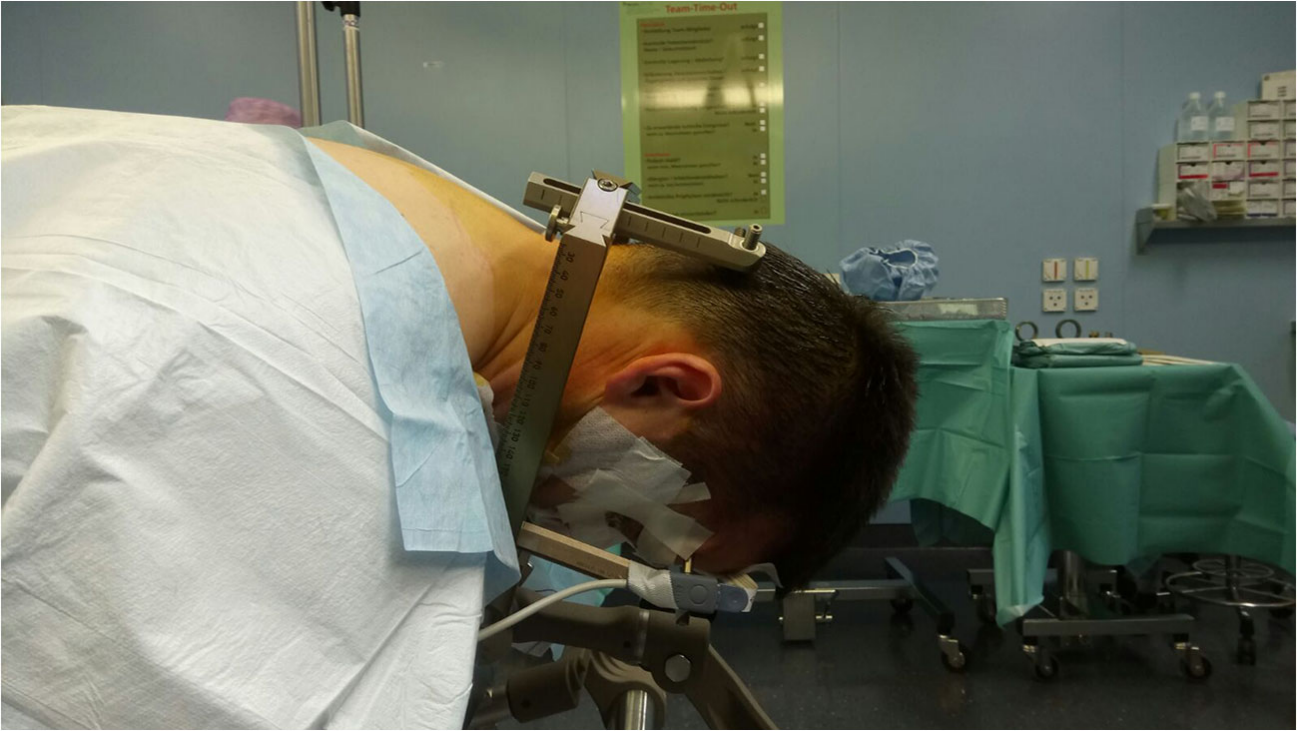
Patient positioning lateral view

**Fig. 3:**
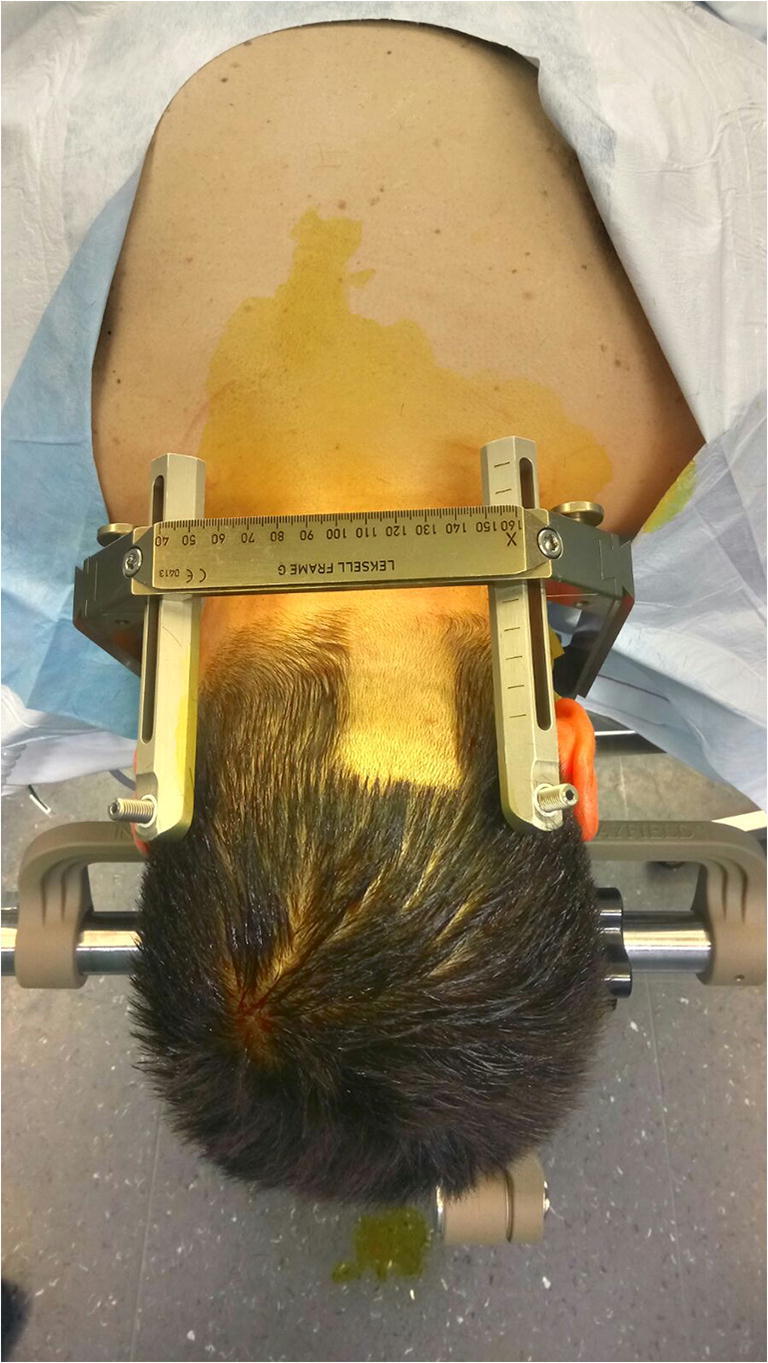
Patient positioning posterior view

**Fig. 4:**
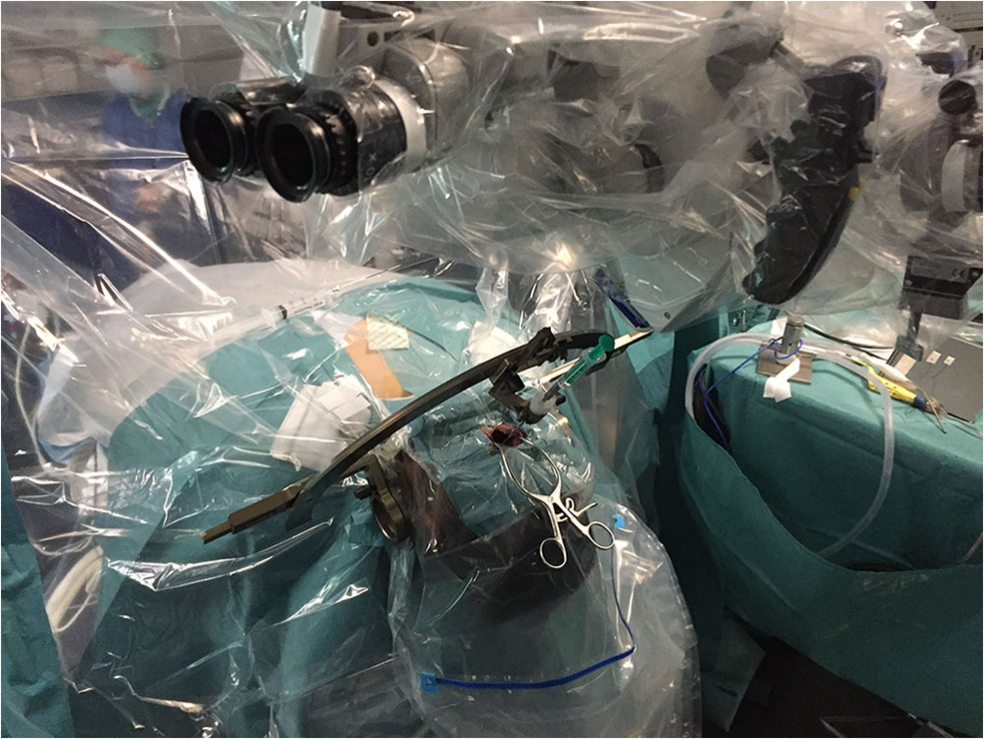
Operation setup: stereotactic frame with the microscope and the biopsy needle in place

The procedure was performed under general anesthesia without intraoperative neuromonitoring. The Leksell stereotactic frame was fitted to the patient’s head. The target and trajectory were defined based on MRI scan. A preoperative stereotactic CT scan was performed and coregistered with the patient’s MRI on a Brainlab work station (Iplan Net 3.0, Brainlab Elements, Germany). The patient was turned into prone position, the head inclined, and the Leksell frame was fixed to the operating table. Hair was shaved paramedially on the left side for a paramedian skin incision (Figs. 2 and 3). Team time out was performed for patient safety. After skin incision, the muscles were dissected with monopolar coagulation and the occipital bone was exposed. A small round craniotomy was performed with a diamond drill, and the bone flap was removed. Under microscopic view, the dura was opened crosswise, the arachnoidea of the cisterna magna was opened, and the arachnoideal adhesions were cut. After identification of the midline of the medulla oblongata, the superficial vessels, and the entry point, the biopsy needle was inserted at the planned target with stereotactic guidance under the microscope view (Fig. 4). The tissue was examined intraoperatively by a neuropathologist, indicating the diagnosis of an ependymoma. Another specimen was taken for the definitive analysis, and the biopsy needle was retracted. Minor bleeding from the tissue was stopped with thorough irrigation. The dura was sutured in a watertight fashion; the bone flap was replaced by palacos, which was fitted to the bone defect and fixed to the skull with mini plates; the muscles were adapted; and the skin was closed. Postoperatively, the patient presented unchanged preexisting symptoms of subtle balance disturbance and occasional double vision with additional singultus, which disappeared after a few days. Histology yielded a tanycytic ependymoma WHO grade II, and the patient underwent adjuvant radiotherapy according to our interdisciplinary neurooncology board.

## Indications

This approach can be proposed when stereotactic biopsy of any lesion of the distal and posterior medulla oblongata is indicated.

## Limitations

There might be limitations regarding the accuracy of the stereotactic biopsy due to brain shift after (1) positioning the patient in prone position whereas stereotactic CT is performed in supine position and (2) CSF leakage after opening the cisterna magna. Furthermore, accurate low positioning of the stereotactic frame is mandatory to be able to reproduce the planned trajectory, perform craniotomy, and place the microscope to get visualization of all the needed anatomical structures.

## How to avoid complications

The posterior approach avoids crossing the whole brainstem (midbrain, pons, and almost whole medulla) before reaching the lesion, which in turn avoids potential devastating complications along the cortico-subcortical and brainstem trajectory. We recommend to perform the stereotactic procedure under direct microscopic view for the following reasons: (1) It allows the opening of the cisterna magna and dissection of the arachnoidal adhesions and the identification and eventual displacement of vessels (PICA branches) along the trajectory to the brainstem. (2) It allows the anatomical exposure of the surface of the medulla oblongata and identification of the midline as well as surface vessels that must be avoided. (3) It allows the precise visualization of the entry point of the needle at the midline of the medulla oblongata, which may be shifted after the opening of the cisterna magna and subsequent CSF leakage, in order to avoid injury of posterior fiber tracts. (4) Because the approach is made through the cisterna magna before reaching the brainstem, it allows for control that the biopsy needle does not push the brainstem instead of penetrating into the tissue.

## Specific perioperative considerations

We performed the surgery under general anesthesia due to prone positioning of the patient on the operating table and also to avoid severe or unsustainable facial pain occurring by the introduction of the biopsy needle in the vicinity of the region of the spinal trigeminal tract [2].

## Specific information to give to the patient about surgery and potential risks

Besides the general risks of stereotactic biopsies, it is important to mention potential lesions of the posterior tracts and the caudal brainstem nuclei, especially the bilateral X nerve nuclei, resulting in severe disabilities or even death. A negative biopsy result due to brain shift is important to mention as well, resulting in another biopsy, which may enhance complication rate.

## Electronic supplementary material

A round suboccipital craniotomy was performed and the bone flap removed. Crosswise opening of the dura, dissection and opening of the arachnoidea. To visualize the entry point under the microscope, the craniotomy needed to be further enlarged with the drill. Exposition of the dorsal medulla and careful introduction of the stereotactic biopsy needle into the medulla oblongata under microscopic view.

ESM 1 (MP4 287563 kb)

ESM 2 (MP4 50662 kb)

## Abbreviations

F.r.: formatio reticularis
G: fasciculus gracilis
Vp: nucleus sensibilis principalis trigemini
X: nuclei vagi
Ts: tractus (and Nucleus tractus) solitarii
Ol.i: oliva inferior
Grpo: griseum pontis
Tcsp: tractus corticospinalis
Ni: substantia nigra
Co.i: colliculus inferior
Co.s: colliculus superior
D/Dm: nucleus dorsomedialis thalami
Hpth: hypothalamus
